# Peak Intraocular Pressure Time during Water Drinking Test and Its Relationship with Glaucoma Severity

**DOI:** 10.18502/jovr.v17i1.10167

**Published:** 2022-01-21

**Authors:** Carolina Nicolela Susanna, Bianca Nicolela Susanna, Fernanda Nicolela Susanna, Remo Susanna Jr, Carlos Gustavo De Moraes

**Affiliations:** ^1^Department of Ophthalmology, ABC Foundation School of Medicine, Santo André, Brazil; ^2^Department of Ophthalmology, University of Sao Paulo School of Medicine, Sao Paulo, Brazil; ^3^Department of Ophthalmology, Columbia University Irving Medical Center, New York, United States

**Keywords:** Glaucoma Severity, IOP Peak Time, Primary Open-angle Glaucoma, Water Drinking Test

## Abstract

**Purpose:**

To investigate the association between the time of occurrence of intraocular pressure (IOP) peaks during the water-drinking test (WDT) and visual field damage in a cohort of primary open-angle glaucoma (POAG) patients.

**Methods:**

In this retrospective, cross-sectional study, 98 eyes from 49 consecutive POAG patients were followed in a referral clinical practice. The relationship between the time when IOP peaks occurred during the WDT and the visual field mean deviation (MD) assessed with 24-2 visual field was tested with mixed-effects models.

**Results:**

MD value was significantly associated with the time of IOP peak occurrence (*P *= 0.020) when adjusting for the number of medications, but not with the IOP peak values (*P = *0.238).

**Conclusion:**

The time of IOP peaks occurrence during the WDT was associated with glaucoma severity among treated POAG patients.

##  INTRODUCTION

Provocative tests have been widely employed in medicine to assess changes in physiological systems when stressed under strenuous conditions. For instance, coronary ischemia, not usually noted in physiologic conditions, may become evident when the subject undergoes a treadmill provocative test or following intravenous pharmacological stimulation. Depending on the magnitude of the change, treatment may be required to prevent long-term complications.

Similarly, the water-drinking test (WDT) is a stress test used to assess intraocular pressure (IOP) behavior and indirectly evaluate the outflow facility of the eye.^[1]^ Glaucoma progression in patients whose IOP is apparently well-controlled during clinic visits maintain a challenge. A satisfactory correlation between clinic-based IOP measurements and mean circadian IOP have been shown, even though not predictive of the peak IOP.^[2]^ In fact, more than 70% of IOP peaks occur at night or in the early morning hours.^[3,4,5,6]^ However, monitoring IOP 24 hr is not practical in routine glaucoma practice. Diurnal tension curves (DTC) misses IOP peaks occurring overnight.^[7]^ The WDT is a reliable and feasible means to estimate peak IOP.

While many glaucomatous eyes may have seemingly controlled IOP during office hours or usual steady-state conditions, IOP peaks triggered by this test may reveal pressure measurements inconsistent with controlled disease and which could yield to disease progression in the long run. In fact, the peak IOP elicited during the WDT has been shown to correlate with the IOP peak that occurs during the day^[8,9,10,11,12]^ and is highly reproducible.^[9,13,14]^ More importantly, it has been shown to be associated with the risk of visual field (VF) progression of glaucoma and disease severity.^[15,16,17,18]^ Recently, it has also been suggested that the WDT could be used to evaluate retinal ganglion cell function and hence have potential application for risk assessment.^[18]^ In addition, the WDT is an indicator of treatment efficacy, assessing the effect of hypotensive drugs as well as surgeries.^[9,19,20,21,22]^

The mechanism of IOP elevation remains unclear, but there are some postulates, such as limited outflow facility, increased episcleral venous pressure (EVP), increased IOP mediated by the autonomic nervous system, and choroidal expansion.^[23,24,25]^ Eyes with lower outflow facility should experience higher IOP peaks after ingestion of water than eyes with normal outflow function, thus being a surrogate measure of the outflow system of the eye and its ability to respond to transient IOP elevation.^[26]^ The time interval in which peak IOP occurs after the ingestion of water can also be related to the ability of the drainage system to maintain IOP homeostasis. Eyes with worse outflow facility may experience continued IOP rise during the WDT, and as so, later IOP peaks than eyes with better outflow facility.

This study aims to investigate the association between severity of glaucomatous VF loss, the magnitude, and the time of IOP peaks during the WDT in a group of treated primary open-angle glaucoma (POAG) patients.

**Table 1:** Baseline characteristics


**Variables**	**Data**
**Number of eyes**	98
**Age**	60 ± 12 (range: 33–95)
**Race** ** Caucasian** ** Asian**	88% 12%
**Sex** ** Female** ** Male **	54% 46%
**Number of medications**	of 2 ± 1 (range: 0–5)
**Latanoprost use **	76 (77.5%)
**Mean baseline MD**	–8.23 ± 7.94 dB (range: –31.19 to 2.38 dB)
**Mean baseline IOP**	14 ± 3 mmHg (range: 8 to 22 mmHg)
**Mean peak IOP**	18 ± 4 mmHg (range: 10 to 30 mmHg)
${}^{*}$ Presented mean ± standard deviation, calculated using summary statistics. MD, mean deviation; IOP, intraocular pressure	

**Table 2:** Distribution of number of eyes, mean MD, and mean IOP peak value of eyes according to the time of IOP peak in the WDT


**Time of IOP peak**	**Number of eyes**	**MD value (dB)** ${}^{†}$	**IOP peak (mmHg)** ${}^{†}$
**15 **	20 (20.4%)	–4.36 ± 5.51	17 ± 4
**30**	42 (42.9%)	–9.35 ± 7.98	19 ± 3
**45**	36 (36.7%)	–9.13 ± 8.56	19 ± 5
${}^{†}$ Presented as mean ± standard deviation, calculated using summary statistics. MD, mean deviation; IOP, intraocular pressure; WDT, water-drinking test.			

**Table 3:** Results of the mixed model evaluating the association between the MD and time of IOP peak, IOP peak value, and number of medications.


**Parameter**	**Coefficient**	**95% CI**	***P*** **-value**
Time of IOP peak	–0.155	–0.284 to –0.025	0.020
Peak value	– 0.237	–0.630 to 0.156	0.238
Number of medications	–1.137	–2.899 to 0.625	0.206
Constant	3.859	–4.691 to 12.411	0.376
${}^{†}$ Calculated using mixed effect model. CI, confidence interval			

**Figure 1:**
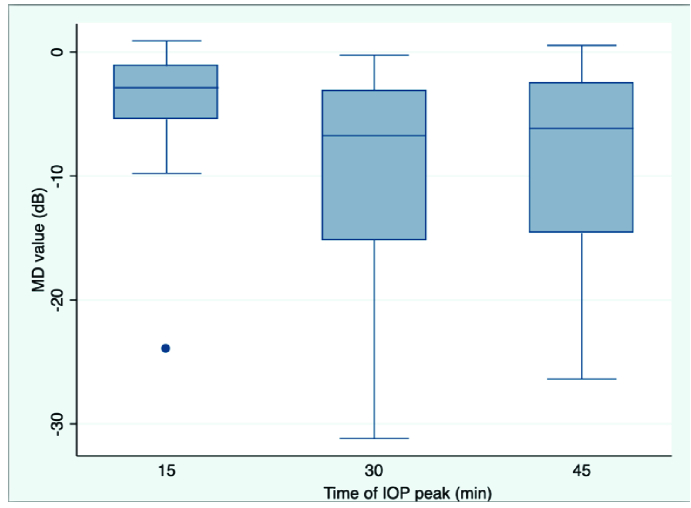
Distribution of mean deviation value at each time point of the WDT. ${}^{†}$ Boxplot depicting the MD distribution. MD, mean deviation; IOP, intraocular pressure; WDT, water-drinking test; min, minutes.

##  METHODS

This retrospective, cross-sectional study included 98 eyes from 49 consecutive POAG patients followed in a referral glaucoma center. The study protocol adhered to the tenets of the Declaration of Helsinki^[27]^ and was approved by the committee of ethics. Informed consent for the research was obtained from all the patients. Consecutive patients that met the inclusion and exclusion criteria were selected for the present study.

A review of medical history, IOP measurement with Goldmann applanation tonometry, best-corrected visual acuity, and slit-lamp biomicroscopy was performed in these patients. Patients were included if they had a glaucomatous appearing optic disc during disc photograph evaluation defined by a senior glaucoma specialist associated with glaucomatous VF loss on 24-2 standard automated perimetry. VF loss was defined according to the modified Anderson's criteria. These results were confirmed on at least two consecutive examinations.

Included eyes had a best-corrected visual acuity of at least 20/40, spherical refraction better than ± 5.00 diopters, and cylinder correction within 3.00 diopters. We excluded participants with non-glaucomatous optic neuropathy, closed or narrow angle assessed by gonioscopic examination, retinal disease, secondary glaucoma, or any other abnormality that could interfere with VF testing. None of the patients had undergone trabeculectomy or laser trabeculoplasty and none had cataract surgery within the last six months before enrollment.

The water-drinking test (WDT) consists of one baseline IOP measurement, followed by ingestion of 800 mL of water in 5 min and three more IOP measurements taken at 15-min intervals.^[28]^ All participants were required to stop liquid ingestion 2 hr before the test. Intraocular pressure measurements were performed with a Goldmann applanation tonometer (Haag-Streit, GmbH, Switzerland). The maximum value of the three measurements was considered as the IOP peak during the WDT.^[9]^ The time of the peak was defined as the time when the maximum IOP was measured. To minimize the effect of the IOP circadian rhythm, all WDT were performed between 4:00 PM and 5:00 PM.

Standard achromatic perimetry was performed with the Humphrey VF Analyzer (24-2 SITA-Standard; Carl Zeiss Meditec Inc., Dublin, CA). All patients underwent VF testing and reliable exams ( < 20% fixation losses, < 33% false-positive and false-negative rates) were analyzed. Visual field tests and WDT were performed up to four months apart.

### Statistical Analyses

Statisticalcomparisons were performed between patients with mixed-effects models, which considers the correlation between both eyes of the same patient.

Statistical analysis was performed using Stata Version 14 (StataCorp LP, College Station, TX). Statistical significance was reached at *P* < 5%.

##  RESULTS 

Ninety-eight eyes from 49 POAG patients were analyzed. The mean age of patients was 60 ± 12 years (range, 33–95) and54% were women. Patients were on a mean of 2 ± 1 (range, 0–5) IOP lowering medications. The mean of mean deviation values (MD) was –8.23 ± 7.94 dB (range, –31.19 to 2.38 dB). Baseline characteristics are described in Table 1.

Table 2 depicts the distribution of the number of eyes, mean MD values (dB), and mean IOP peak according to the time point of the WDT in which the IOP peak occurred. The mean IOP peak and the mean MD values (17 ± 4 mmHg and 4.36 ± 5.51 dB, respectively) were lower at 15 min compared to 30 min (19 ± 3 mmHg and –9.35 ± 7.98 dB) and 45 min (19 ± 5 mmHg and –9.13 ± 8.56 dB) in the WDT.

The box plot of the distribution of MD value in each time IOP peak occurrence (Figure 1) shows lower MD values in the later time points (30 and 45 min) compared to 15 min.

Separate multivariable models showed a statistically significant relationship between the time of IOP peak and MD values (*P *= 0.010) adjusting for number of medications. However, peak value was not associated with MD values when adjusting for number of medications (*P *= 0.117).

The results of the mixed-effect model relating MD values to the time of IOP peak, IOP peak value, and number of medications together are presented in Table 3. Eyes with more damage in VFs had later IOP peaks during WDT (*P *= 0.020). Neither number of medications nor IOP peak value were significantly related to MD values (*P = *0.238 and *P *= 0.206, respectively).

##  DISCUSSION 

Intraocular pressure peak is a key risk factor for glaucoma progression.^[29,30,31]^ To better investigate other parameters obtained from the WDT, we tested whether the IOP peak time was related to the level of glaucomatous functional damage, which might reflect the eye's outflow system status of a given eye. Therefore, it is expected that in eyes with worse outflow facility, IOP elevation may remain rising for longer time, leading to later IOP peaks during the WDT.

Razeghinejad et al^[32]^ investigated the effect of WDT after tube shunt surgery and trabeculectomy and showed that 30 min after the WDT, IOP in the trabeculectomy group initiated to decline, whereas for the tube shunt group it remained increasing up to 60 min, which might have implications on tubes' efficacy in advanced glaucoma patients. Additionally, Waisbourd et al^[22]^ investigated the effect of the WDT on the IOP of patients with angle-closure glaucoma and demonstrated that after peripheral iridotomy was performed, patients had a more pronounced IOP recovery, probably due to an increased trabecular meshwork area exposure following treatment. This corroborates the reasoning that eyes with impaired outflow have different time responses during the WDT.

We found that the time during WDT of IOP peaks' occurrence was associated with glaucoma severity in a population with treated POAG. Specifically, eyes with more severe disease had a later IOP peak than eyes with less severe disease (*P *= 0.020). In other words, eyes with later IOP peaks experienced continued IOP rise during the WDT until the maximum IOP was reached (IOP peak) and as so, a longer period of IOP elevation than eyes with earlier IOP peaks, possibly reflecting a better ability of these eyes to handle transient IOP elevation.

Accordantly, De Moraes et al^[33]^ showed that the number of long peaks assessed with contact lens sensor (CLS) was the best predictors of faster progression in treated glaucoma patients.^[33]^

In contrast with results found by other authors,^[15,16,17]^ there was no association between peak IOP value and MD (*P *= 0.238) in our study. Probably because patients were under treatment based on physician's discretion, which was adjusted to reduce IOP peaks elicited by the WDT. Therefore, patients showing more advanced glaucoma were likely prone to receive aggressive therapy in both eyes to achieve lower target IOP peaks.

One limitation is that this was a retrospective study. In order to reduce selection bias, we consecutively selected patients from a cohort in which all patients had routinely been submitted to the WDT.

Further prospective studies evaluating these WDT parameters, preferably with patients free of topical treatment, should be done to better understand the relationship between the peak time and VF defect.

In conclusion, this study demonstrated that the time of occurrence of IOP peak measured with the WDT was associated with glaucoma severity and might be an additional tool to evaluate glaucomatous patients.

##  Financial Support and Sponsorship

Nil.

##  Conflicts of Interest

There are no conflicts of interest.
